# Estimation of mortality rate ratios for chronic conditions with misclassification of disease status at death

**DOI:** 10.1186/s12874-023-02111-3

**Published:** 2024-01-03

**Authors:** Sabrina Voß, Annika Hoyer, Sandra Landwehr, Meda E. Pavkov, Edward Gregg, Ralph Brinks

**Affiliations:** 1 Chair for Medical Biometry and Epidemiology, Faculty of Health, Witten/Herdecke University, Witten, Germany; 2 Biostatistics and Medical Biometry, Medical School EWL, Bielefeld University, Bielefeld, Germany; 3 Regional Association of Statutory Health Insurance Physicians, Strategic Data Analysis Unit, Düsseldorf, Germany; 4 Division of Diabetes Translation, National Center for Chronic Disease Prevention and Health Promotion, Centers for Diseases Control and Prevention, Atlanta, GA USA; 5 Department of Epidemiology and Biostatistics, School of Public Health, Imperial College London, London, UK; 6 German Diabetes Center, Institute for Biometry and Epidemiology, Düsseldorf, Germany

**Keywords:** Missing disease information, Illness-death model, Biased mortality rate ratio, Simulation study, Estimation of mortality rate ratio

## Abstract

Estimation of mortality rates and mortality rate ratios (MRR) of diseased and non-diseased individuals is a core metric of disease impact used in chronic disease epidemiology. Estimation of mortality rates is often conducted through retrospective linkage of information from nationwide surveys such as the National Health Interview Survey (NHIS) and death registries. These surveys usually collect information on disease status during only one study visit. This infrequency leads to missing disease information (with right censored survival times) for deceased individuals who were disease-free at study participation, and a possibly biased estimation of the MRR because of possible undetected disease onset after study participation. This occurrence is called “misclassification of disease status at death (MicDaD)” and it is a potentially common source of bias in epidemiologic studies. In this study, we conducted a simulation analysis with a high and a low incidence setting to assess the extent of MicDaD-bias in the estimated mortality. For the simulated populations, MRR for diseased and non-diseased individuals with and without MicDaD were calculated and compared. Magnitude of MicDaD-bias depends on and is driven by the incidence of the chronic disease under consideration; our analysis revealed a noticeable shift towards underestimation for high incidences when MicDaD is present. Impact of MicDaD was smaller for lower incidence (but associated with greater uncertainty in the estimation of MRR in general). Further research can consider the amount of missing information and potential influencers such as duration and risk factors of the disease.

**Supplementary Information:**

The online version contains supplementary material available at 10.1186/s12874-023-02111-3.

## Introduction

Chronic diseases are a major burden for health care systems worldwide. As an example, in 2019, 37.1 million people in the US already had diagnosed or undiagnosed diabetes and 1.4 million new cases were detected [1]. Epidemiological studies allow estimation of the extent of impact of chronic diseases on a population’s health with calculation of prevalence, incidence, and mortality rates. For example, many nationally representative longitudinal follow-up studies in the US estimate mortality rates by linking surveys, such as the National Health Interview Survey (NHIS) and National Health and Nutrition Examination Study (NHANES) [2] to information from death registries (for example, the National Death Index in the USA [3]), retrospectively. Due to the design, these studies collect information on a participant’s disease status at one time point only. As a result, information may be missing about a diagnosis after study participation. For example, a deceased individual who had been disease-free at study participation might have developed the (chronic) disease (without recovery) afterwards. These individuals with undetected disease onset are misclassified as non-diseased at death. Therefore, mortality estimates based on these data are potentially biased. This leads to misclassification of disease status at death (MicDaD) for the estimation of mortality rate ratios (MRR).

Binder et al. have investigated existence, extent, and impact of misclassification of disease status on the estimation of hazards on risk factors [4]. Moreover, Binder et al. [5] found that 46.4% of (prospective) cohort studies with time to event endpoint used assessment of disease information at follow-up visits only and conventional analysis of data had the potential of being biased because of unclear disease status at death. Moreover, a simulation study revealed that this bias leads to under- or overestimation of the impact of risk factors on hazards [5]. Despite the common use of these approaches, the quantitative impact of MicDaD on estimates of (excess) mortality and the extent and the direction of this bias has not yet been examined systematically. The aim of our study was to measure and evaluate the extent of MicDaD on MRR and how it is influenced by the incidence and to assess this bias through a simulation study in a high incidence setting (based on type 2 diabetes) and a low incidence setting (based on lupus erythematosus).

First, we will give a detailed description of the problem of misclassification of disease status at death and the illness-death model (IDM). Then, we will explain the structure of our simulation study and the methods we used for the analysis of populations with and without misclassification of disease status at death. After the presentation of the results of our simulation study, we will summarize and review study results and limitations.

## Methods

### Misclassification of disease status at death

**Fig. 1:**
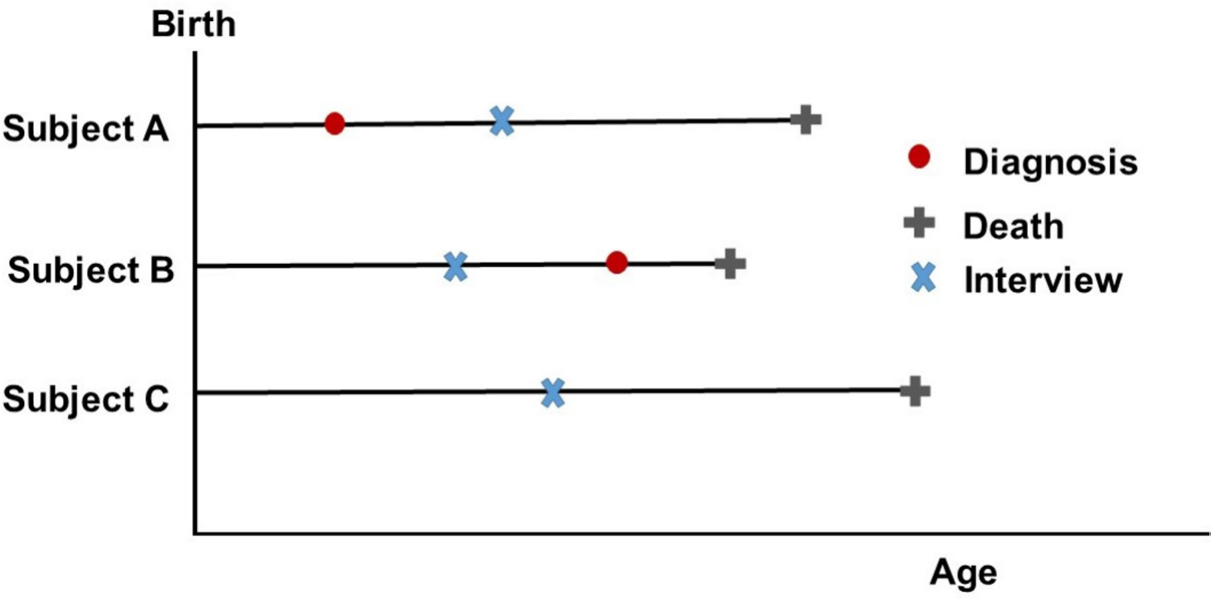
Schematic description of the problem of misclassification of disease status at death. Individual A has the correct classification of disease status as diagnosis took place before the survey; individual B has misclassification of disease status at death as diagnosis happened after survey; and individual C has correct classification as they are disease-free until death

Information on a population’s disease status can be collected in cross-sectional interview-based surveys. As these surveys are conducted only at discrete time points, the problem of censored information of disease status arises for individuals who are disease-free at interview. Assuming that all individuals are interviewed only once in their lifetime, disease status is collected at one fixed date and will not be updated in the resulting database afterwards. A person who is disease-free at study baseline can develop the chronic disease of interest (without recovery) between study participation and death; thus, the classification as disease-free could be incorrect, leading to MicDaD. Therefore, estimation of MRR for diseased and non-diseased individuals could potentially be biased, caused by false or missing information on disease status of deceased persons. The classification as healthy would only be correct for individuals that remained disease-free until the date of death. The true disease status at death, based on the survey, can only be obtained without doubt for individuals already diseased at interview. The possible scenarios are depicted in Fig. 1.

Individual A was diagnosed (red dot) with the chronic disease of interest before the date of survey-participation (blue cross). This person will be correctly classified as having the disease at death. Individual C is disease-free at the date of study participation and stays disease-free (according to the chronic disease of interest) until death. Therefore, this individual is correctly classified as disease-free at death. Individual B is disease-free at the interview but develops the chronic disease under consideration between survey participation and death. Consequently, when information on disease status (at death) is collected from the survey, individual B will be incorrectly classified as disease-free at death.

### Illness-death model

**Fig. 2:**
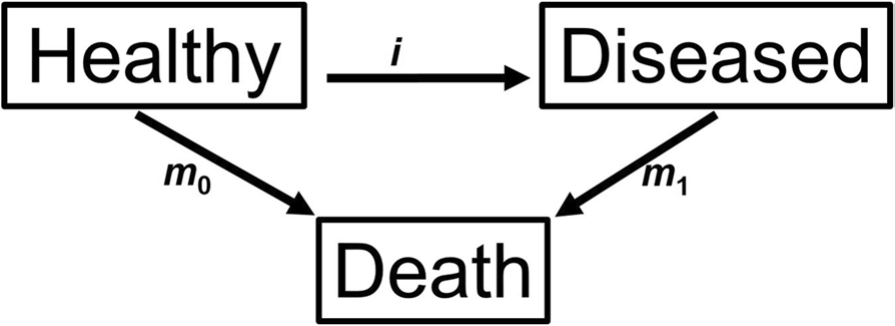
Illness-death model with three different states: healthy, diseased, and death, and three transition rates: mortality rate of the non-diseased *m*_0_, mortality rate of the diseased *m*_1_ and incidence rate *i*

The simulation study is based on the illness-death model, a multi-state model with three possible states: Healthy (from chronic disease of interest), Diseased, and Death and three transition rates: mortality rates and incidence rate. Figure 2 shows a schematic description of the illness-death model.

The IDM describes a closed population according to a (chronic) disease. Every individual is disease-free at the beginning and starts in the healthy state. All individuals in the population experience at least one transition. The mortality rates of diseased (*m*_1_) and non-diseased (*m*_0_) individuals and the incidence rate (*i*) are the age dependent transition intensities between the states of the IDM. All individuals in the population examined will either directly move from healthy to death along *m*_0,_ or transit according to *i* from healthy to diseased first and then die in the diseased state with *m*_1_. We use an illness-death model where only two ways of transition are possible and we assume a (chronic) disease without recovery (remission) from disease state [6]:

1. A healthy individual directly transits from the healthy state to the death state (without transiting to the disease state) according to the mortality rate of non-diseased *m*_0_ (one transition in the IDM).
2. A healthy individual contracts the chronic disease at some age (depending on the underlying incidence rate of the disease) and transits from the healthy state into the disease state. All individuals in the disease state remain in this state (no remission) until they have their second transition from disease state into the death state (mortality rate of diseased *m*_1_) [7].

In general, mortality and incidence rates are dependent on (calendar-) time *t* and age *a*. To simplify the structure and analysis of our simulation study, we simulated the population as a birth cohort with all individuals born in the same year. As a result, we have a time-heterogenous illness-death model that depends on age as the only timescale.

### Simulation of populations

**Fig. 3:**
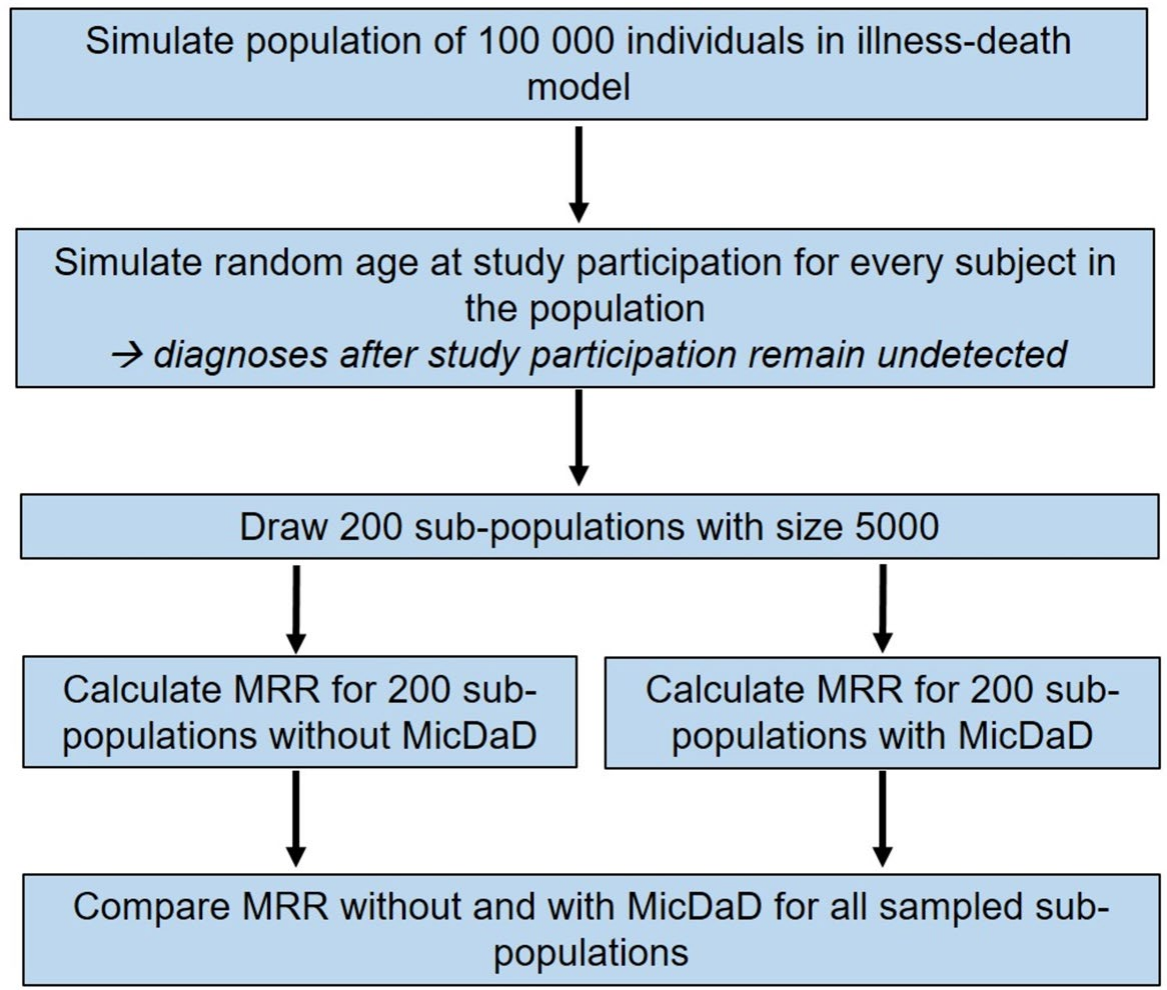
Flow-chart of the simulation study

Figure 3 shows the steps of the simulation study presented in this article.

The population in the illness-death model used in our study with a size of 100 000 individuals was simulated in two steps with discrete event simulation for the high (based on type 2 diabetes) and the low incidence setting (based on lupus erythematosus). As a discrete event simulation is a microsimulation, we simulated event times (ages of transition in the IDM: change of state) for every subject individually and collected date of birth, age at diagnosis (age_diagnosis_), and age at death (age_death_). At the beginning, we simulated the age of first transition in the IDM—either from the healthy state to death state or from the healthy state to disease state. For all individuals with diagnosis at first failure time, a second time for death (diseased) was simulated. A detailed description of discrete event simulation, including algorithms and distribution functions of first and second failure time, can be found in Brinks et al. (2014) [8]. The age at diagnosis was set to NA for individuals that were disease-free at death. Data of the simulated population was stored with age at entering the state, duration in each state, age at leaving the state, and information about the entering state. For more information and a detailed description of this Lexis data format used in the R package Epi, see Carstensen (2021) [9] and Carstensen and Plummer (2011) [10].

### Parameter settings for simulation of populations

For the simulation of a high incidence setting (motivated by type 2 diabetes) and a low incidence setting (motivated by lupus erythematosus) with discrete event simulation, we used the mortality and incidence rates taken from the references summarized in Table 1. The exact formulas for the transition rates between the states are in our Supplementary material.

**Table 1:** Description of parameter settings for simulation of populations with discrete event simulation method

	Setting	
	High incidence	Low incidence
**Chronic disease**	Type 2 diabetes	Lupus erythematosus
**Incidence and mortality rates**	Brinks (2016) [7]	Brinks et al. (2016) [11]
**Age range (years) considered**	40 to 80	40 to 80

### Simulation of study participation

In the second part of our simulation, we added a random age at survey participation (age_survey_). The age of survey participation was generated individually from a uniform distribution, ranging from 18 to 110 years of age. We set the lower age limit to exclude children from the surveys; and we determined the upper limit to ensure that every individual is in the death state at the end of the simulation study. Using the age at survey participation, we could mimic misclassification of disease status at death; diagnoses after study participation (age_survey_ < age_diagnosis_) remained undetected. All individuals with undetected disease were (wrongly) categorized as disease-free at death (as the transition from healthy to diseased was not documented) which leads to a misclassification of true disease status at death. These individuals would be included as healthy in the calculation of mortality rate ratios, even though they contracted the chronic disease.

### Sampling of sub-populations

In the third part of our study, we evaluated extent and direction of the bias in the estimation of mortality rates and the MRR caused by misclassification of disease status at death. Bias assessment was based on 200 sampled populations with 5000 individuals that were drawn from the original simulated population of 100 000 individuals. The number of populations (*n* = 200) was chosen as a compromise between reasonable estimation of the distributions and run-time of the simulation (in order not to increase the current already long term even further). For every sampled sub-population, a data set with and without misclassification was conducted, resulting in two sets for every sampled sub-population.

### Evaluation of mortality rate ratio

A Poisson model with log-link function and age of individuals as the only independent variable was used to estimate age-dependent mortality rates of diseased and non-diseased individuals for every sub-population (with MicDaD: *m*_0dis_,*m*_1dis_ and without MicDaD: *m*_0_,*m*_1_). Age-dependent MRRs with and without misclassification of disease status at death in the sub-populations at specific ages were calculated via *m*_0_(*a*) / *m*_1_(*a*). Comparison was performed based on median MRRs in the sub-populations without and with MicDaD (supported by 2.5%- and 97.5%- quantiles to display the 95% range of estimated MRR). Kernel density was estimated for calculated MRRs without and with MicDaD and displayed in a graph with additional rug plots to compare location and variability of MRRs. As every sampled sub-population was evaluated without and with MicDaD, differences of MRR with and without MicDaD for every population at different ages were calculated. The closer to zero the difference between MRR with and without MicDaD, the lower the impact of MicDaD on MRR. Bias was calculated as the median of the differences with and without MicDaD with a value bigger than 0 indicating an overestimation of MRR with MicDaD and a value smaller than 0 showing an underestimated MRR in the presence of MicDaD.

## Results

### Simulated populations

#### High incidence setting

The simulated population in the high incidence setting consisted of 100 000 individuals. Median age at diagnosis of the chronic disease within the high incidence setting was 60.75 years and median age at death was 75.85 years for diseased and 74.00 years for non-diseased individuals (overall median age at death was 74.84 years). Table 2 displays the transitions between the states in the illness-death model. In the high incidence setting, 40 019 diagnoses were reported.

**Table 2:** Transitions in the illness-death model in the simulated population with 100 000 individuals (high incidence setting)

		**To**	
		**Disease**	**Death**
**From**	**Healthy**	40 019	59 981
	**Disease**	0	40 019

**Fig. 4:**
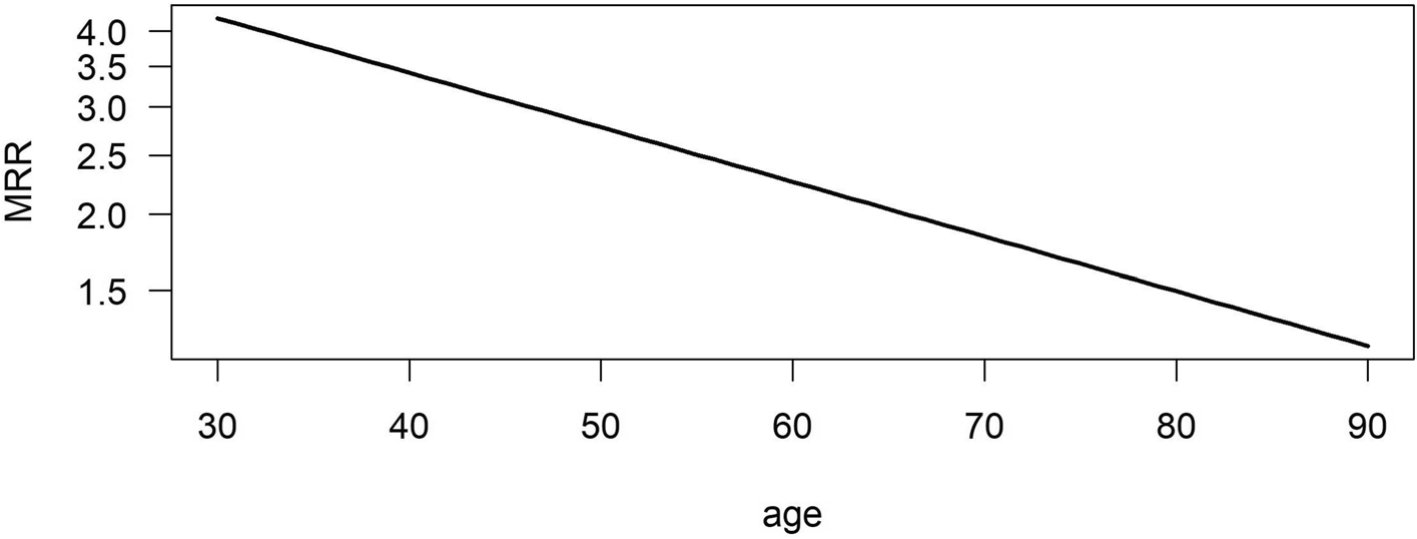
Age-specific mortality rate ratio in the simulated population (high incidence setting)

Figure 4 presents the decreasing age-dependent MRRs on a log-scale in the population with MRR 3.42 at age 40 years, 2.26 at age 60 years, and 1.50 at age 80 years, respectively.

#### Low incidence setting

The second population resulting from an illness-death model with a low incidence setting consisted of 100 000 individuals with 196 transitions from the healthy to the disease state (see Table 3). Median age at diagnosis was 32.55 years, median age at death was 63.14 years for diseased and 70.53 years for non-diseased subjects (overall: 70.52 years).

**Table 3:** Transitions in the illness-death model in the simulated population with 100 000 individuals (low incidence setting)

		**To**	
		**Disease**	**Death**
**From**	**Healthy**	196	99 804
	**Disease**	0	196

**Fig. 5:**
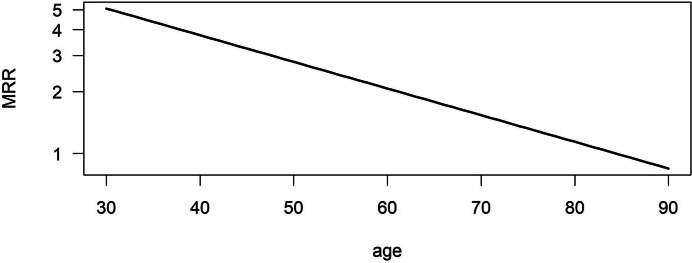
Age-specific mortality rate ratio in the simulated population in the low incidence setting

Figure 5 shows the age-dependent MRRs for individuals in the low incidence setting. MRR for the ages 40 years, 60 years and 80 years were 3.93, 2.30 and 1.35, respectively.

### Estimated mortality rate ratios in sampled sub-populations

#### High incidence setting

**Fig. 6:**
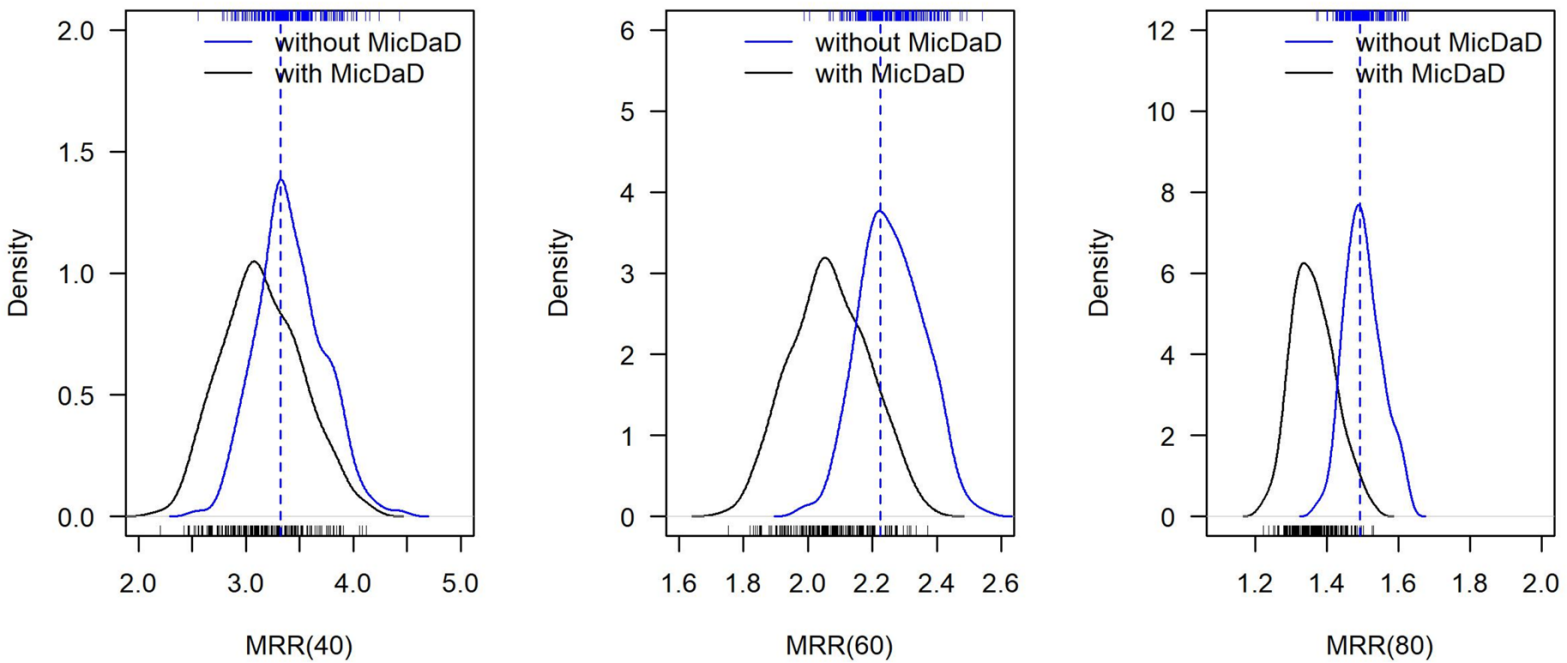
Distribution of mortality rate ratios (MMRs) at ages 40, 60, 80 years: input rate ratio for simulation (dashed line), MRR without MicDaD in blue, and MRR with MicDaD from 200 sampled populations in black (high incidence setting)

Figure 6 shows the distribution of the estimated MRRs in the 200 populations as small bars at the x-axis and with kernel density estimates in the sampled sub-population at ages 40, 60, and 80 years without as blue line and with MicDaD as black line. Table 4 summarizes these values with medians and 2.5%- and 97.5%- quantiles.

**Table 4:** True input MRR for simulation and medians (and 2.5%, 97.5% quantile) in simulated population and across 200 sampled sub-populations (without and with MicDaD) (high incidence setting)

**Age**	**40 years**	**60 years**	**80 years**
**True MRR (input for simulation)**	3.32	2.23	1.49
**MRR without MicDaD**	3.38 (2.87–4.05)	2.26 (2.08–2.44)	1.50 (1.42–1.61)
**MRR with MicDaD**	3.11 (2.52–3.88)	2.06 (1.84–2.30)	1.36 (1.26–1.49)

As the mortality rate ratios depend on age, differences between MRRs without and with MicDaD changed according to age of individuals (see Table 4). For example, the true input-mortality rate ratio in the high incidence setting was 3.32 at the age of 40 years, 2.23 at age 60 years, and 1.49 at age 80 years. MRRs in the simulated population with 100 000 individuals were close to these input values with 3.42 (age 40 years), 2.26 (age 60 years) and 1.50 (age 80 years).

The median MRR in the sampled sub-populations without MicDaD at the age 40 years was 3.38 and close to the input MRR, whereas the median MRR in the populations with MicDaD showed a shift towards smaller values of MRRs in the sub-populations with median MRR 3.11. For other ages (for example 60 years or 80 years) we could also detect smaller MRRs in the sub-populations with MicDaD with a shift towards 1 (with 1 indicating no difference in the mortality rates of diseased and non-diseased individuals). Consequently, we saw that having populations with MicDaD led to an underestimation of the actual MRR for all ages (with smaller difference and smaller mortality rate ratios at higher ages).

Table 5 shows the bias in the estimation of MRR in case of MicDaD as the median of the differences.

**Table 5:** Bias of estimation of MRR in 200 sampled sub-populations without and with MicDaD including 2.5% and 97.5% quantiles and frequencies of over- and underestimation of MRR (high incidence setting)

**Age**	**40 years**	**60 years**	**80 years**
**Bias**	-0.27 (-0.76–0.27)	-0.20 (-0.34 – -0.04)	-0.14 (-0.22 – -0.04)
**MRR overestimated with MicDaD**	35 ( 17.5%)	0 ( 0%)	0 ( 0%)
**MRR underestimated with MicDaD**	165 ( 82.5%)	200 ( 100%)	200 ( 100%)

At age 40 years most ages were smaller or equal to 0. At ages 60 years and 80 years all differences were smaller or equal to 0. This shows that nearly all MMRs in the sub-populations were too small compared to the real MRRs in the same population without having MicDaD. MRRs in the high incidence setting were underestimated when some transitions from healthy to disease remain unseen and are not included in estimation of mortality rates. Table 5 displays bias of the estimation of MRR due to MicDaD only.

#### Low incidence setting

**Fig. 7:**
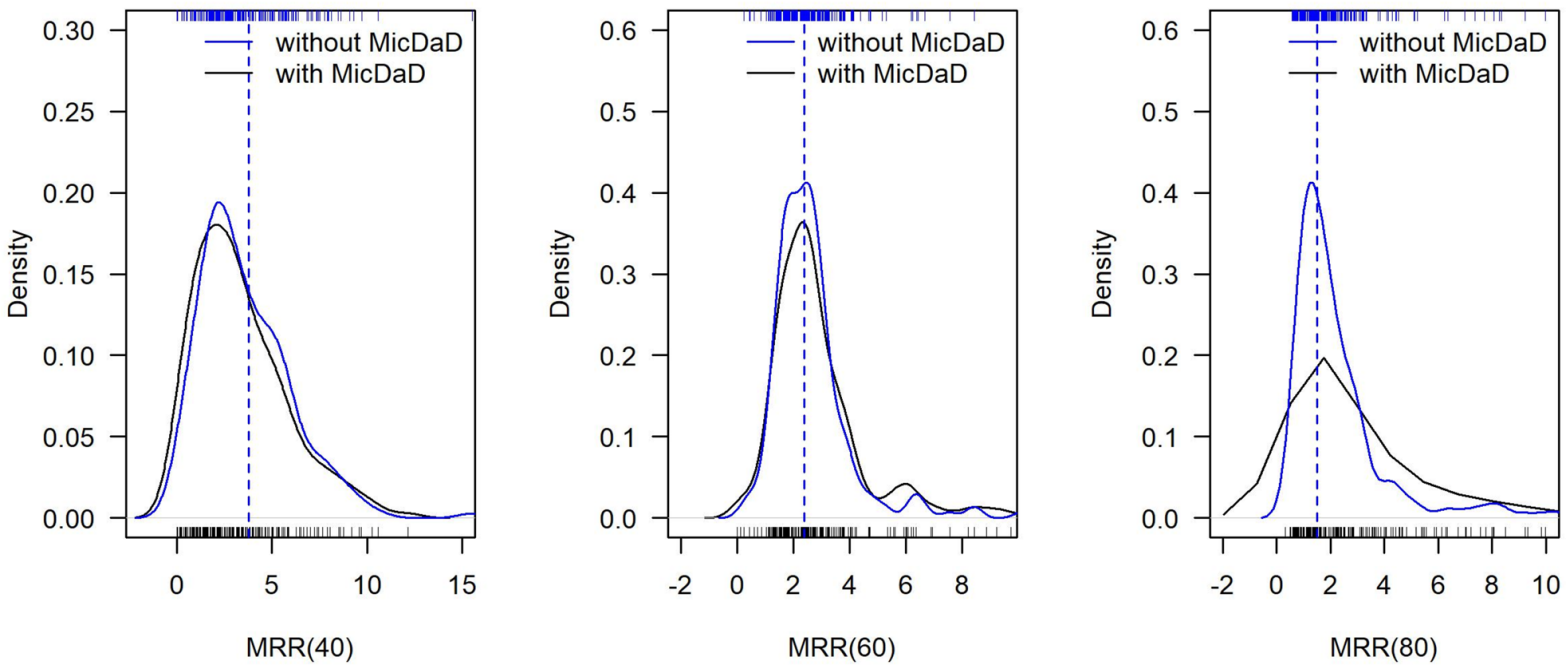
Distribution of mortality rate ratios (MMRs) at 40, 60, 80 years: input rate ratio for simulation (dashed line), MRR without MicDaD in blue and MRR with MicDaD from 200 sampled populations in black (low incidence setting)

Figure 7 shows the distribution of MRR (bars at the x-axis) with kernel density estimates without (blue) and with (black) MicDaD in the 200 sampled sub-populations in the low incidence setting at ages 40 years, 60 years, and 80 years. This figure shows that distributions of MRRs without and with MicDaD were mostly overlapping. This indicates only little impact of MicDaD in estimating MRR in this low incidence setting. At higher ages (60 years) the median in the sub-populations without MicDaD differed slightly from the median MRR in the sub-populations with MicDaD (2.42 vs. 2.49). For ages 40 years and 60 years, differences in medians were greater and showed a shift towards 1. At age 80 years uncertainty was higher potentially because median age at death was 63.14 years for diseased and 70.53 years for non-diseased subjects. Table 6 has these values including medians and 2.5%- and 97.5%- quantiles.

**Table 6:** True input MRR for simulation and medians (and 2.5%, 97.5% quantile) in simulated population and across 200 sampled sub-populations (without and with MicDaD) (low incidence setting)

**Age**	**40 years**	**60 years**	**80 years**
**True MRR (input for simulation)**	3.79	2.39	1.51
**MRR without MicDaD**	3.18 (0.40–9.07)	2.42 (1.03–6.71)	1.76 (0.64–23.88)
**MRR with MicDaD**	2.91 (0.04–9.21)	2.49 (0.71–9.85)	2.27 (0.58–113.09)

The exact intra-differences of the MMRs in every sub-population with and without MicDaD at ages 40 years, 60 years and 80 years are displayed in Table 7 as the bias of the estimation in the 200 sampled sub-populations caused by MicDaD including 2.5% and 97.5% quantiles in the low incidence setting.

**Table 7:** Bias of estimation of MRR in 200 sampled sub-populations without and with MicDaD, including 2.5% and 97.5% quantiles and frequencies of over- and underestimation of MRR (low incidence setting)

**Age**	**40 years**	**60 years**	**80 years**
**Bias**	-0.05 (-2.69–1.75)	0.00 (-1.03–7.33)	0.00 (-1.11–107.85)
**MRR overestimated with MicDaD**	70 ( 35%)	89 ( 44.5%)	99 ( 49.5%)
**MRR underestimated with MicDaD**	106 ( 53%)	87 ( 43.5%)	77 ( 38.5%)

At all ages about half of the differences were greater and half of the differences were smaller than 0. This shows that both underestimation as well as overestimation of MRR occurred in the sub-populations when there was MicDaD. Therefore, MicDaD showed less and a non-systematic impact on the estimation of MRR in our low incidence setting.

## Discussion

In this article, we showed that the impact and extent of misclassification of disease status at death is driven by incidence of the chronic disease of interest.

### Summary

Cross-sectional interview-based surveys can be used for the collection of information on the existence of chronic diseases in a population. However, if these interviews are used to obtain information about an individual’s disease status at death, misclassification is possible because study participants are interviewed only once and surveys do not capture that some disease-free participants may develop a chronic disease before death. We conducted a simulation study to assess the extent and direction of this possible misclassification bias and how this bias is influenced by the incidence of the chronic disease of interest. Therefore, our simulation study was based on a high incidence (type 2 diabetes) and a low incidence disease (lupus erythematosus) with populations consisting of 100 000 individuals that were transiting through an illness-death model with Healthy, Diseased and Death (final state for every individual) as possible states. A total of 200 sub-populations with 5000 individuals were randomly drawn from these populations for each chronic disease. For every individual, a random age of study participation was simulated; diagnosis at an age greater than this age remained unseen with a misclassification as non-diseased. For every population, MRR was evaluated without and with possible misclassification of disease status at death (MicDaD). We compared median (with 2.5% and 97.5% quantiles) MRRs in the simulated populations without and with MicDaD. Misclassification of disease status at death led to underestimated MRRs for chronic diseases with a high incidence (such as type 2 diabetes). For low-incidence chronic disease (such as lupus erythematosus) MicDaD caused lower to no bias in the estimation of MRR. This was the first study that investigated the impact of misclassification of disease status at death on MRRs.

### Interpretation

Analysis of simulated data in the high incidence setting showed a gap between MRR of populations without and with MicDaD. Populations with MicDaD had smaller values with higher differences at younger ages and smaller values of MRR when MicDaD occurred. As the values of MRR shifted towards 1, underestimation of the MRR was detected in the high incidence setting with misclassification of disease status at death for some individuals in a population. Since extent or number of undetected diagnoses is unknown in practice, it is possible that bias caused from MicDaD is larger than in the simulations and settings considered in our study. Particularly in the case of chronic diseases with high incidences (and a mortality similar to that used in the presented simulation study), a serious underestimation of the risk of death caused by or with a chronic disease is to be expected. Results in the low incidence setting (based on incidence and mortality rates for lupus erythematosus) lead to the suggestion that for a chronic disease with a low incidence, MicDaD has less impact. But still MRR is underestimated with values shifting towards 1. Bigger differences between MicDaD and no MicDaD and greater uncertainty in the low incidence setting (for example at age 80 years: without MicDaD: 1.76 with 2.5–97.5% quartiles: [0.64–23.88] vs. with MicDaD: 2.27 with 2.5–97.5% quartiles: [0.58–113.09]) are potentially caused by low mortality at that age and low incidence in general. In addition to that, differences in bias between younger and older ages (40 and 60–80) were recognized that were potentially caused by the age-dependency of MRR and incidence. Nevertheless, further studies could explore this more detailed.

Our analysis showed that misclassification of disease status at death has an impact on the estimation of mortality rates and MRRs for chronic diseases (especially for chronic diseases with higher incidences). MicDaD leads to an underestimation of the MRR of diseased and non-diseased individuals. This underestimation results in a misinterpreted risk of death with chronic diseases.

### Limitations

Our study is the first work to examine the influence of misclassification of disease status at death on the estimation of MRR, but there are limitations.

First, the current analysis only considers age at entry and exit into states in the IDM. The duration in a state remained unconsidered in our analysis and in the estimation of mortality rates and the related MRR. Duration influences mortality and should be considered in future research.

A second limitation is that we neither controlled nor systematically evaluated the amount of missing information on disease status at death. A random age for one-time participation in the interview was determined and compared to age at diagnosis to decide whether information on diagnosis remained unseen. In further analyses, the amount of missing “diagnoses” could be systematically considered and varied to capture susceptibility to bias in the estimate of the MRR, depending on the amount of missing diagnoses. As a possible solution to this problem, simulation of age at study participation could be performed based on different statistical distributions, or on a distribution based on the actual ages at study participation in studies like NHIS. This approach can be used to systematically analyze whether or to what extent age at study participation influences bias caused by MicDaD. Additionally, it can be examined how the amount of missing information about disease status at death influences the magnitude of this misclassification bias. It is possible that higher ages at study participation and a smaller amount of missing information could reduce the bias caused by MicDaD. However, further research is needed.

We simulated age-dependent mortality rates only. In contrast to Binder et al. (2014) [4] which considered constant transition intensities, the age-dependency of the transition rates in the IDM is a strength of our study because real life incidence and mortality are dependent on age. But in practice, more (risk) factors, such as sex and social economic position, influence mortality and could be considered in future research.

A fourth limitation of our simulation study is population size. We only simulated one population with 100 000 individuals and sampled sub-populations with 5000 individuals. It is possible that extent of bias caused by MicDaD can differ in bigger or smaller populations. As the population size possibly influences the estimation of mortality rates, further simulation studies can be used to investigate the impact of population size on the bias in estimating MRR with MicDaD.

We considered only two different scenarios in our analysis. Thus, the analysis was based on only two possible combinations of age-dependent incidence and mortality rates (based on real-world data from type 2 diabetes and lupus erythematosus). Further analyses are needed to obtain a systematic analysis of the susceptibility and the extent of a bias by MicDaD in relation to mortality and incidence. For this reason, more and different scenarios with varying mortality and incidence rates and combinations of these may be needed.

A fifth limitation is that we reduced misclassification of disease status at death to a problem with one timescale only (age of individuals) and an ordinary differential equation.

Because it is possible that other times, such as year (calendar time), also influence the MicDaD-induced extent of bias in estimating the MRR, further research that includes a description of transitions in the IDM is needed. Furthermore, partial differential equations with age and calendar-time as time scales (see Brinks et al. (2016) [12]) may be conducted.

In the current simulation, individuals are surveyed (interview) only once in their lifetime. However, it is possible that study participants can be interviewed more than once. Additional analyses with the possibility of repeated study participation could be conducted.

In order to achieve a more detailed description of the extent of missing information of disease status at death, we plan to perform further research considering risk factors, other scenarios with varying mortalities and incidences, and different amounts of missing information and population sizes. Additionally, we will add a second timescale (calendar-time) to our research and consider the possibility of repeated study participation and duration in a state in the IDM.

An additional limitation is that we only used 2 settings (high incidence with later age at onset; low incidence with earlier age at onset) based on the age-depedent incidence (and thus indirect regulation of the extent of MicDaD) for the simulation. A wider range of incidence settings would allow a more detailed analysis of different possible situations and frequencies of MicDaD and give a more diverse picture of the effects of MicDaD. However, the aim of our work was a first time description of this important epidemiological phenomenon that potentially plays a role in many studies. The effect of MicDaD (direction and magnitude of the effect of this misclassification) in studies with this design was completely unknown until now. Our goal was no comprehensive simulation study, but to gain a first insight into this complex topic.

### Comparative literature

In contrast to our analysis of mortality rates, Binder et al. (2014) [4] performed a study on the extent of bias in estimating the hazards of risk factors. As they did not evaluate bias on estimation of mortality, the study is less comparable to our evaluation. Binder et al. evaluated four scenarios with different transition rates in the IDM (mortality rates and incidence rate) and three risk factors with varying impact on these transitions and our study did not investigate risk factors. A second difference to our study was that Binder et al. (2014) assumed mortality and incidence rates to be constant over time. The authors found no impact of misclassification of disease status (and therefore no bias caused by misclassification) on the estimation of hazards for risk factors when mortality rates for diseased and non-diseased individuals were the same and constant over time. These results cannot be compared to our results as we neither had constant nor identical mortality rates for healthy and diseased individuals. In settings with differing mortality rates, the authors found that bias was growing for higher fractions of missing disease information. Bias in estimation of hazards for risk factors was dependent on the constellation of mortality and incidence rates; for higher mortality of healthy individuals the effect of a risk factor is overestimated while it was underestimated for high incidence rates. In our analyses we saw a comparable tendency in the estimation of mortality rates as MRR was underestimated in the high incidence setting with MicDaD. An important limitation of the study from Binder et al. (2014) was that they had constant hazards only (transition rates in the IDM); although, mortality is known to be age-dependent.

Another study by Binder et al. [4] investigated how unknown disease status leads to over- and underestimation of effect size estimates for risk factors. Moreover, they revealed that nearly half of all prospective cohort studies are at risk of this bias, especially when data analysis is performed using standard methods instead of methods they describe in their publication [13].

Another concept of bias in observational studies is the immortal time bias (ITB) that is possible in epidemiological studies when a treated (exposed) and a non-treated (non-exposed) group are compared. Individuals in the treated group are immortal before study participation [14]. ITB overestimates treatment effect on death whereas MicDaD underestimates mortality caused by the chronic condition of interest without consideration of any treatment.

So the difference between MicDaD and ITB is that MicDaD is caused by diseased individuals that are falsely treated as healthy when having the outcome ‘death’, whereas ITB concentrates on diseased individuals with known diagnosis that are unable to die. Additionally, death is impossible for an individual with ITB but with MicDaD individuals die without diagnosis. Therefore, results of studies analysing ITB are less comparable to our study.

## Conclusion

The problem of misclassification of disease status at death influences the estimation of MRR. Impact and extent of MicDaD is driven by the incidence of the chronic disease of interest. In a high incidence setting MRR was underestimated, whereas in a setting with low-incidence, chronic disease MicDaD caused lower to no bias in the estimation of MRR.

### Supplementary Information

**Additional file 1.**

## Data Availability

All data analysed is simulated. The resulting (simulated) datasets and source code (for simulation and anaylsis) generated and analysed during the current study are available in the Zenodo repository, [with DOI 7661148].
