# Supplementary material for: Estimation of mortality rate ratios for chronic conditions with misclassification of disease status at death
Source: BMC Med Res Methodol. 2024 Jan 3;24:2. doi: 10.1186/s12874-023-02111-3 (PMC10765798; doi:10.1186/s12874-023-02111-3)
Supplement: Supplementary file 1 — Additional file 1. [file 12874_2023_2111_MOESM1_ESM.docx]

**Supplementary Material**

**Transition Rates for the simulation in IDM**

**High incidence setting (based on type 2 diabetes)**

Mortality rate of non-diseased

$$m_{0}\left( a \right)= exp(-11+0.11\cdot a)$$

Mortality rate of diseased

$$m_{1}\left( a \right)= exp(-9+0.09\cdot a)$$

Incidence rate

$$i\left( a \right)= \frac{a-30}{2000} , a>30$$

The related integrals used in discrete event simulation were:

$$M_{0}\left( a \right)= \frac{exp\left( -11+0.11\cdot a \right)-exp(-11)}{0.11}$$

$$M_{1}\left( a \right)= \frac{exp\left( -9+0.09\cdot a \right)-exp(-9)}{0.09}$$

$$I\left( a \right)= \frac{(a-30)\cdot(a-30)}{4000} , a>30$$

**Low incidence setting (based on Lupus erythematodes)**

Mortality rate of non-diseased

$$m_{0}\left( a \right)= exp(-9.3+0.093\cdot a)$$

Mortality rate of diseased

$$m_{1}\left( a \right)= exp\left( \log\left( 6 \right)-\log\left( 6 \right)-log(1.2)\cdot((a-20)/70) \right)\cdot exp(-9.3+0.093\cdot a)$$

Incidence rate

$$i\left( a \right)= 9.083e^{-6}-6.758e^{-13}\cdot a^{5}+1.213e^{-10}\cdot a^{4}-7.169e^{-9}\cdot a^{3}+1.346e^{-7}\cdot a^{2}+6.637e^{-7}\cdot a)$$

The related integrals used in discrete event simulation were:

$M_{0}\left( a \right)= 10.75\cdot exp(-9.3)\cdot(\exp\left( 0.093\cdot a \right)-1)$

$$M_{1}\left( a \right)= 14.28\cdot exp(-7.05)\cdot(\exp\left( 0.07\cdot a \right)-1)$$

$$I\left( a \right)= 9.083e^{-6}\cdot a-(6.758e^{-13}/6)\cdot a^{6}+(1.213e^{-10}/5)\cdot a^{5} -(7.169e^{-9}/4)\cdot a^{4} +(1.346e^{-7}/3)\cdot a^{3}+(6.637e^{-7}/2)\cdot a^{2})$$
